# Polypharmacy in Older Adults Undergoing Major Surgery: Prevalence, Association With Postoperative Cognitive Dysfunction and Potential Associated Anesthetic Agents

**DOI:** 10.3389/fmed.2022.811954

**Published:** 2022-02-15

**Authors:** Saranya Lertkovit, Arunotai Siriussawakul, Patumporn Suraarunsumrit, Wanicha Lertpipopmetha, Natapong Manomaiwong, Wittachi Wivatdechakul, Varalak Srinonprasert

**Affiliations:** ^1^Department of Anesthesiology, Faculty of Medicine Siriraj Hospital, Mahidol University, Bangkok, Thailand; ^2^Faculty of Medicine Siriraj Hospital, Integrated Perioperative Geriatric Excellent Research Center, Mahidol University, Bangkok, Thailand; ^3^Division of Geriatric Medicine, Department of Medicine, Faculty of Medicine Siriraj Hospital, Mahidol University, Bangkok, Thailand; ^4^Department of Anatomy, Faculty of Medicine Siriraj Hospital, Mahidol University, Bangkok, Thailand; ^5^Department of Internal Medicine, Faculty of Medicine, Khon Kaen University, Khon Kaen, Thailand; ^6^Department of Orthopedics, Hatyai Hospital, Hat Yai, Thailand

**Keywords:** prevalence, polypharmacy, postoperative cognitive dysfunction, older adult patients, elective major surgery

## Abstract

**Background:**

Polypharmacy, which is defined as the use of 5 or more medications, can exert significant adverse impact on older adult patients. The objective of this study was to determine the prevalence of polypharmacy, and to investigate its association with postoperative cognitive dysfunction (POCD) in older adult patients who underwent elective major surgery at Siriraj Hospital—Thailand's largest national tertiary referral center.

**Methods:**

This prospective study included older adult patients aged ≥65 years who were scheduled for elective major surgery during December, 2017 to December, 2019 study period. Patient demographic, sociodemographic, anthropometric, clinical, comorbidity, anesthetic, surgical, and medication data were collected and compared between the polypharmacy and non-polypharmacy groups. Postoperative cognitive dysfunction (POCD) was diagnosed in patients with at least a 2-point decrease in their Montreal Cognitive Assessment score after surgery. Multivariate logistic regression analysis was used to identify independent predictors of POCD.

**Results:**

A total of 250 patients (141 males, 109 females) with an average age of 72.88 ± 6.93 years were included. The prevalence of polypharmacy was 74%. Preoperative data showed the polypharmacy group to be more likely to be receiving potentially inappropriate medications, to be scheduled for cardiovascular thoracic surgery, and to have more comorbidities. There was a non-significant trend in the association of polypharmacy and POCD (crude odds ratio (OR): 2.11, 95% confidence interval [CI]: 0.90–4.94; *p* = 0.08). Benzodiazepine, desflurane, or isoflurane administration during surgery were all significantly associated with POCD in univariate analysis. Multivariate analysis revealed intraoperative benzodiazepine (adjusted OR [aOR]: 2.24, 95% CI: 1.10–4.68; *p* = 0.026) and isoflurane (aOR: 2.80, 95% CI: 1.35–5.81; *p* = 0.006) as two independent variables associated with the development of POCD. Desflurane was found to be a protective factor for POCD with a crude OR of 0.17 (95% CI: 0.03–0.74, *p* = 0.019); however, independent association was not found in multivariate analysis.

**Conclusion:**

There was a high prevalence of polypharmacy in this study; however, although close (*p* = 0.08), significant association was not found between polypharmacy and POCD. Benzodiazepine and isoflurane were both identified as independent predictors of the development of POCD among older adult patients undergoing elective major surgery, especially among those classified as polypharmacy.

## Introduction

An aging society is defined as >10% of the population aged over 60 years, and an aged society is defined as more than 14% of the population aged over 60 years (1). Most high-income countries and many middle-income countries (including Thailand) have become aging countries. Increasing age is commonly associated with more comorbidities, multiple medications, and deterioration of organ function. Alterations of drug pharmacokinetics and pharmacodynamics among older adults increases the risk of adverse drug reactions, subsequent hospitalization, and increased mortality (2).

Polypharmacy is a global problem that is expected to worsen with advances in medicine and the increasing development and discovery of new drugs. There are several definitions of polypharmacy in the literature, and many studies that reported significant association between polypharmacy and subsequent negative clinical outcomes. The most commonly reported factors are taking five or more prescribed drugs and receiving potentially inappropriate medications (PIMs) (3–5). The likelihood of adverse drug reactions increases commensurate with the number of drugs taken, and rises in adverse drug reactions results in an increased number of hospital admissions (6). Polypharmacy exerts several other detrimental effects on older adults, including delirium and cognitive impairment, which increase medical expenses, morbidity, and mortality (7, 8). The risk increases if one or more prescriptions in a polypharmacy case are drugs defined as PIMs.

As the population ages, there is an increased incidence of anesthesia and surgery among older adults, and it has been established that age-related factors increase the likelihood of postoperative complications (9). Furthermore, polypharmacy was found in the majority of older adult patients undergoing major elective noncardiac surgery, and it was found to be associated with a reduced survival rate and a higher rate of adverse events (10).

Postoperative cognitive dysfunction (POCD) is defined as a decline in cognitive function after anesthesia and surgery. Symptoms of POCD may develop from within 1 week after surgery to months after surgery. In this setting, cognitive function includes learning and memory, verbal ability, perception, attention, executive function, and abstract thinking (11). Meanwhile postoperative delirium (POD) is a sudden change in mental status marked by a disturbance of awareness of the environment and a disruption in attention after surgery. There are three types of POD expression: hypoactive, hyperactive, and mixed. POD may also be involved in the development of POCD. The difference between POD and POCD is that POD diagnosis involves symptom detection, whereas POCD diagnosis requires neuropsychological testing administered pre-and post-operatively. The prevalence of POCD was reported to be as high as 41% among older adult patients after some surgical procedures (12). Understanding the relationship among anesthesia, surgery, and cognitive impairment is essential for guiding clinical practice (13). Several potential causes of POCD have been proposed, including advanced age, prior delirium, preoperative cognitive impairment, low education, and the use of anticholinergic medications prior to surgery (14–18). Data specific to the association between POCD and medications used during the perioperative period are scarce. Previous study has addressed the issue of polypharmacy and cognitive decline, but not in postoperative setting (19). Accordingly, the aim of this study was to determine the prevalence of polypharmacy, and to investigate its association with POCD in older adult patients who underwent elective major surgery at Siriraj Hospital—Thailand's largest national tertiary referral center.

## Methods

This prospective study was conducted as part of the Siriraj Integrated Perioperative Geriatric Research Network of the Faculty of Medicine Siriraj Hospital, Mahidol University, Bangkok, Thailand (study COA No. 515/2017). We recruited Thai-speaking patients who were aged 65 years or older and scheduled for elective major surgery during the December, 2017 to December, 2019 study period. Major surgery was defined as a procedure potentially lead to organ ischemia, high intraoperative blood loss, high noradrenalin requirements, long operative time, and requiring perioperative blood transfusion. Operation generates systemic inflammatory response and the need for intermediate or intensive care was also considered as a major surgery. Potential consequences of major surgery include high morbidity and mortality (20). Patients unable to undergo cognitive assessments, having severe visual or auditory dysfunction, having significant psychotic disorders affecting their ability to cooperate, having preoperative delirium, or being bedridden were excluded. We also excluded patients who were unable to attend follow-up visits during the postoperative period. Patient baseline characteristics and intraoperative data were obtained from their electronic medical records.

### Data Collection

Patient demographic data, comorbidities, body mass index (BMI), and current patient medication data were collected preoperatively via patient interview and review of hospital medical records. Intraoperative and postoperative data were also gathered from medical records, including type of surgery, anesthetic technique, anesthetic medications, operative time, and intraoperative and postoperative complications. Polypharmacy was defined as the use of five or more medications preoperatively (3). PIMs were identified according to Beers criteria (5) and assessment by a geriatrician. The Montreal Cognitive Assessment (MoCA)–Thai version was administered by a psychologist to measure each patient's baseline cognitive status before undergoing surgery. It evaluates at eight different cognitive abilities: visuospatial/executive, naming, memory, attention, language, abstraction, delayed recall, and orientation. The maximum MoCA score is 30, with a higher value indicating better performance. The test has been widely used to assess older people with various form of cognitive dysfunction.

During postoperative days 5–9, the same psychologist visited all enrolled participants at the inpatient ward. If they were medically stable (e.g., absence of acute stroke or hypotension), the MOCA test was readministered. POCD was diagnosed according to the previously published recommendation (21) that POCD be defined as a postoperative decrement of cognitive test scoring ≥1 standard deviation. However, in the present study, we diagnosed POCD when there was decrease of ≥2 points in the postoperative MoCA score compared to the preoperative MoCA score, which was also a previously reported diagnostic method (22).

### Sample Size Calculation and Statistical Analysis

The sample size calculation took into account both components of our study objective. The primary objective of this study was to determine the prevalence of polypharmacy. Previous study reported a prevalence of polypharmacy of approximately 29% (23). Cochran's formula was used to calculate the sample size, with the Z value set to 1.96 at a 95% confidence level and the acceptable tolerance (e) set at 8%. Using those estimates, a minimum sample size of 116 patients would be required to satisfy the primary objective. Sample size calculation for secondary objective was also performed for identification of factors associated with POCD using multivariate analysis. We estimated following previous study which discovering 5 factors independently associated with the development of POCD (24). Using the rule of thumb method suggested for 10 events per variable with estimated prevalence of POCD of 20% from the Siriraj Integrated Perioperative Geriatric Research Network database, a minimum of 250 patients would be required. The final sample size for the study was therfore chosen to be 250.

All data analyses were performed using SPSS Statistics version 18 (SPSS, Inc., Chicago, IL, USA). Participant dermograhic, clinical, and intraoperative data were analyzed using descriptive statistics. We divided patients into two groups according to the number of medications they were taking, as follows: the polypharmacy group (five or more drugs) and the non-polypharmacy group (less than five drugs). Categorical data were compared using chi-square test or Fisher's exact test, and the results are given as number and percentage. Continuous data were compared using 2-sample *t*-test for normally distributed data (results shown as mean plus/minus standard deviation), and using Mann-Whitney *U* test for non-normally distributed data (results show as median and range; minimum, maximum). To identify association between polypharmacy and POCD, we divided participants into groups based on the presence or absence of POCD. Evaluated variables with a *p* <0.10 in univariate analysis were entered into multivariate analysis to identify factors that independently predict POCD. The results of the univariate and multivariate analyses are presented as crude and adjusted odds ratios (respectively) and their respective 95% confidence intervals. A *p* < 0.05 was considered statistically significant for all tests.

## Results

**Figure 1:**
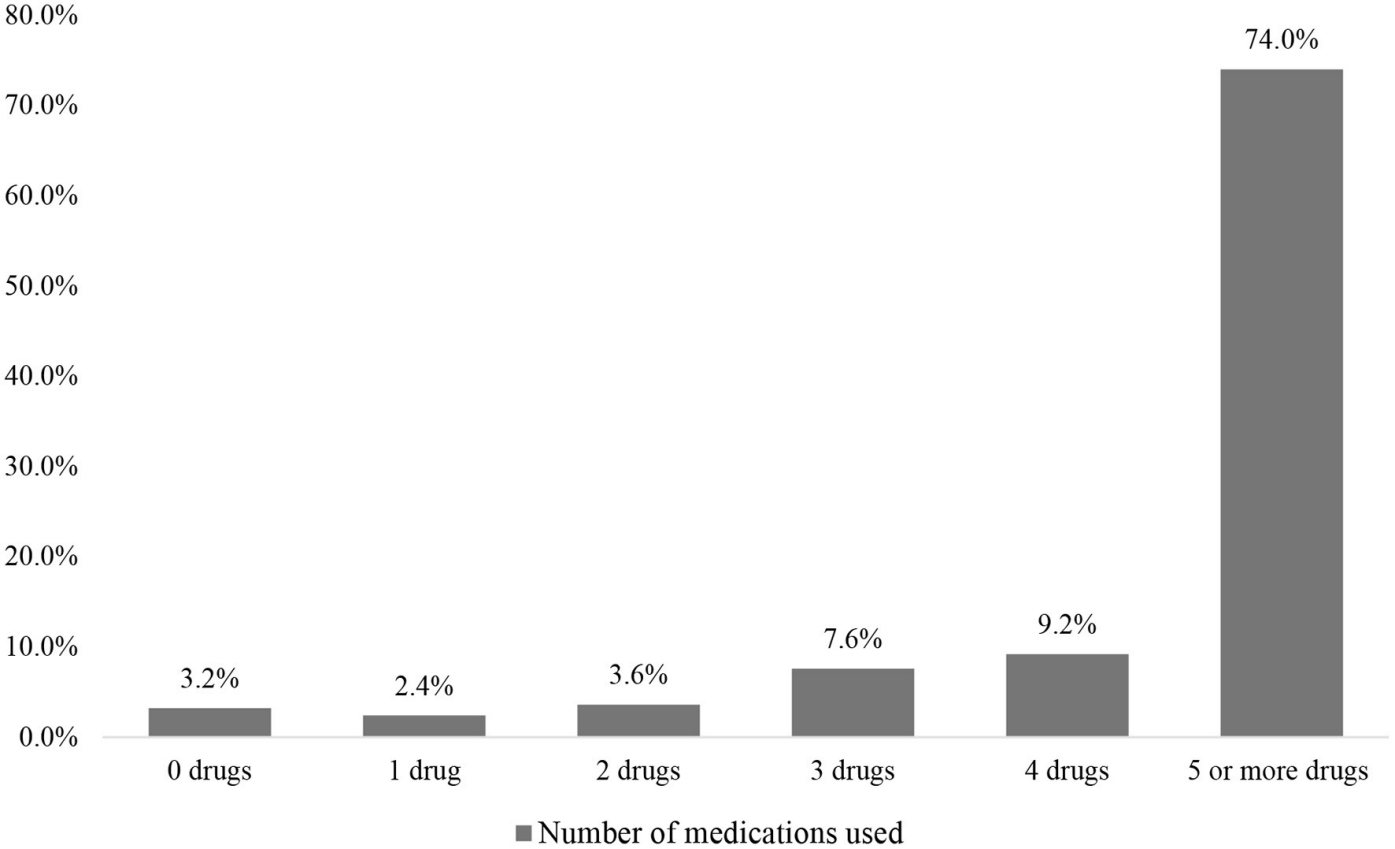
The percentage of overall patients stratified by the number of drugs used. The results show the prevalence of polypharmacy (five or more drugs) to be 74% among older adults who underwent major surgery.

We evaluated 250 participants aged 65 and over who underwent elective major surgery. This study included 141 males and 109 females with an average age of 72.88 ± 6.93 years. Concerning the type of surgery, 141 patients underwent cardiovascular thoracic (CVT) surgery, and 109 had non-CVT surgery (Table 1). As demonstrated in Figure 1, polypharmacy was found in 74% (*n* = 185) of older adult patients who underwent surgery. Moreover, a 31% prevalence of PIMs was found in the polypharmacy group, which was significantly higher than the rate in the non-polypharmacy group (12.3%) (*p* = 0.002).

**Table 1:** Preoperative characteristics of the study population.

**Characteristics**	**(*N* = 250)**
Age (years)	72.88 ± 6.93
<70 years	75 (30.0%)
70–79 years	130 (52.0%)
≥80 years	45 (18.0%)
**Gender**	
Male	141 (56.4%)
Female	109 (43.6%)
**Education**	
<12 years of education	219 (88.7%)
≥ 12 years of education	28 (11.3%)
BMI	24.08 ± 4.10
Underweight (<18.5 kg/m^2^)	24 (9.6%)
Normal (18.5–24.9 kg/m^2^)	131 (52.4%)
Overweight (25–29.9 kg/m^2^)	75 (30.0%)
Obese (≥30.0 kg/m^2^)	20 (8.0%)
**ASA classification**	
II	60 (24.0%)
III	174 (69.6%)
IV	16 (6.4%)
**Site of surgery**	
CVT	141 (56.4%)
Non-CVT	109 (43.6%)
Presence of polypharmacy	185 (74%)
Presence of PIMs	67 (26.8%)

*Data presented as mean ± standard deviation or number and percentage*.

*SD, standard deviation; BMI, body mass index; ASA, American Society of Anesthesiology; CVT, cardiovascular thoracic surgery; PIMs, potentially inappropriate medications*.

**Figure 2:**
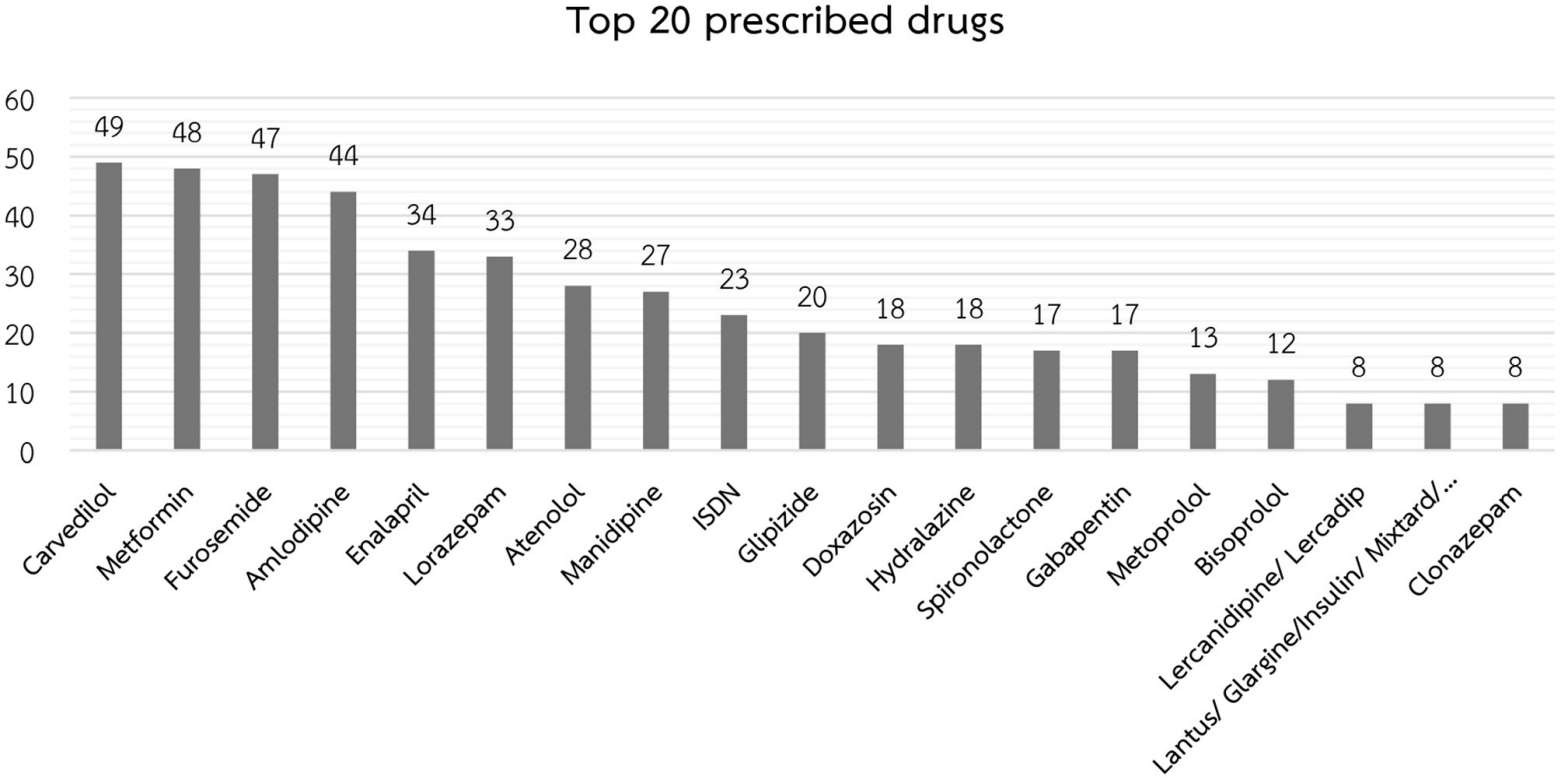
The top 20 most frequently prescribed drugs in the polypharmacy group (results shown as the number of patients out of the 185 patients in the polypharmacy group).

Patients in the polypharmacy group were significantly more likely to be scheduled for CVT surgery, to have higher disease burden (ASA Physical Status classification of three or above), and to have a higher prevalence of comorbidities, including hypertension, dyslipidemia, and ischemic heart disease/myocardial infarction. Antiarrhythmic drugs, antihypertensive drugs, diabetic drugs, diuretic drugs, and a benzodiazepine-based anxiety reliever were among the most common medications prescribed. In contrast, patients in the non-polypharmacy group had significantly more malignant cancer and alcohol use (Table 2). Carvedilol (26.4%), metformin (24.9%), furosemide (25.4%), amlodipine (23.7%), and enalapril (18.4%) were the first five most commonly prescribed drugs for patients in the polypharmacy group (Figure 2). Concerning anesthesia-related variables, which are shown in Table 3, patients with polypharmacy were significantly more likely to receive general anesthetic procedures (82.7%), inhalation (86.5%), isoflurane inhaler (35.1%), a muscle relaxant with rocuronium (42.2%), analgesic with morphine (55.1%), and blood transfusion (66.9%).

**Table 2:** Preoperative characteristics compared between the non-polypharmacy and polypharmacy groups.

**Characteristics**	**Non-polypharmacy (*n* = 65)**	**Polypharmacy (*n* = 185)**	***p*-value**
Age	73.32 ± 6.99	72.73 ± 6.92	0.557
<70 years	14 (21.5%)	61 (33.0%)	0.223
70–79 years	38 (58.5%)	92 (49.7%)	
≥80 years	13 (20.0%)	32 (17.3%)	
Gender			0.697
Male	38 (58.5%)	103 (55.7%)	
Female	27 (41.5%)	82 (44.3%)	
Education			0.424
<12 years of education	55 (85.9%)	164 (89.6%)	
≥ 12 years of education	9 (14.1%)	19 (10.4%)	
ASA classification			<0.001^*^
II	35 (53.8%)	25 (13.5%)	
III	29 (44.6%)	145 (78.4%)	
IV	1 (1.5%)	15 (8.1%)	
**Comorbidities**			
Hypertension	32 (49.2%)	167 (90.3%)	<0.001^*^
Atrial fibrillation	7 (10.8%)	25 (13.5%)	0.569
Congestive heart failure	2 (3.1%)	25 (13.5%)	0.020^*^
Ischemic heart disease/myocardial infarction	9 (13.8%)	112 (60.5%)	<0.001^*^
Valvular heart disease	12 (18.5%)	46 (24.9%)	0.293
Peripheral vascular disease	0 (0.0%)	6 (3.2%)	0.344
Dyslipidemia	32 (49.2%)	138 (74.6%)	<0.001^*^
Hyperthyroid	1 (1.5%)	2 (1.1%)	1.000
Hypothyroid	0 (0.0%)	6 (3.2%)	0.344
Diabetes mellitus	8 (12.3%)	83 (44.9%)	<0.001^*^
Asthma	1 (1.5%)	3 (1.6%)	1.000
COPD	2 (3.1%)	3 (1.6%)	0.607
CKD stage ≥3a	24 (36.9%)	82 (44.3%)	0.299
Malignancy	26 (40.0%)	40 (21.6%)	0.004^*^
Cirrhosis	1 (1.6%)	5 (2.7%)	0.598
Alcohol use	5 (7.7%)	4 (2.2%)	0.040^*^
Current smoker	1 (1.5%)	3 (1.6%)	1.000
**Medication group**			
Antiarrhythmic drug	17 (26.2%)	109 (58.9%)	<0.001^*^
Antidepressant drug	1 (1.5%)	10 (5.4%)	0.297
Antiemetic drug	0 (0.0%)	5 (2.7%)	0.331
Antihypertensive drug	39 (60.0%)	172 (93.0%)	<0.001^*^
Benzodiazepine	3 (4.6%)	41 (22.3%)	<0.001^*^
Diabetic drug	4 (6.2%)	67 (36.4%)	<0.001^*^
Diuretic	10 (15.4%)	58 (31.4%)	0.015^*^
Site of surgery			<0.001^*^
Non-CVT	41 (63.1%)	68 (36.8%)	
CVT	24 (36.9%)	117 (63.2%)	
Presence of PIMs	8 (12.3%)	59 (31.9%)	0.002^*^

*Data presented as mean ± standard deviation or number and percentage*.

*A p < 0.05 indicates statistical significance*.

*ASA, American Society of Anesthesiologists; COPD, chronic obstructive pulmonary disease; CKD, chronic kidney disease; CVT, cardiovascular thoracic surgery; PIMs, potentially inappropriate medications*.

* *p < 0.05*.

**Table 3:** Intraoperative data compared between the non-polypharmacy and polypharmacy groups.

**Data**	**Non-polypharmacy (*n* = 65)**	**Polypharmacy (*n* = 185)**	***p*-value**
Choice of anesthesia			<0.001^*^
GA and RA	17 (26.1%)	21 (11.4%)	
GA	36 (55.4%)	153 (82.7%)	
RA	12 (18.5%)	11 (5.9%)	
**Special monitoring**			
BIS	2 (3.1%)	5 (2.7%)	1.000
NIRS	4 (6.2%)	11 (5.9%)	1.000
Benzodiazepine use	27 (41.5%)	102 (55.1%)	0.059
Dexmedetomidine use	9 (13.8%)	19 (10.3%)	0.440
Intraoperative adverse events	33 (51.6%)	75 (42.6%)	0.218
**Induction agents**			
Thiopental	2 (3.1%)	3 (1.6%)	0.607
Propofol	43 (66.2%)	141 (76.2%)	0.113
Etomidate	1 (1.5%)	3 (1.6%)	1.000
Propofol TCI	7 (10.8%)	14 (7.6%)	0.423
Inhalation use			0.004^*^
No	19 (29.2%)	25 (13.5%)	
Yes	46 (70.8%)	160 (86.5%)	
**Inhalation type**			
Desflurane	13 (20.0%)	29 (15.8%)	0.422
Sevoflurane	25 (38.5%)	66 (35.9%)	0.688
Isoflurane	8 (12.3%)	65 (35.1%)	<0.001^*^
**Muscle relaxant for intubation**			
Pancuronium	0 (0.0%)	0 (0.0%)	NA
Atracurium	13 (20.0%)	29 (15.7%)	0.422
Cis-atracurium	25 (38.5%)	63 (34.1%)	0.522
Succinylcholine	2 (3.1%)	1 (0.5%)	0.167
Rocuronium	13 (20.0%)	78 (42.2%)	<0.001^*^
**Analgesia used**			
Morphine	18 (27.7%)	102 (55.1%)	<0.001^*^
Pethidine	1 (1.5%)	2 (1.1%)	1.000
Fentanyl	59 (90.8%)	179 (96.8%)	0.084
Ketamine	0 (0.0%)	3 (1.6%)	0.570
COX-2 inhibitor	1 (1.5%)	2 (1.1%)	1.000
Reversal agents used^a^	30 (46.2%)	48 (26.4%)	0.003^*^
Blood product used	30 (46.2%)	121 (66.9%)	0.003^*^
**Other complications**			
Hypertension	1 (1.6%)	4 (2.2%)	1.000
Hypotension	29 (44.6%)	67 (37.0%)	0.302
Hypotension^b^	3 (4.7%)	4 (2.2%)	0.382
Severe arrhythmia	9 (13.8%)	29 (16.0%)	0.677
Anesthetic time (min)	289 (40, 610)	282 (53, 775)	0.715

*Data presented as number and percentage or median and range (minimum, maximum)*.

*A p <0.05 indicates statistical significance*.

a *Reversal of muscle relaxant at the end of surgery*.

b *Hypotension requiring continuous intravenous inotropic/vasopressor support*.

*GA, general anesthesia; RA, regional anesthesia; BIS, bispectral index; NIRS, near-infrared spectroscopy monitoring; Propofol TCI, target-controlled infusion of propofol; COX, cyclooxygenase*.

* *p < 0.05*.

Regarding evaluation for POCD in this study. Only 175 patients were available for MoCA testing after surgery. Reason for data missing were unstable medical conditions death, unwillingness to answer questions, and discharging from the hospital prior to evaluation. Of those, 51 individuals (29.1%) were identified as having POCD. Table 4 shows variables potentially associated with POCD compared between the non-POCD and POCD groups. Ischemic heart disease/myocardial infarction, receiving antihypertensive drug, CVT surgery, received benzodiazepine during surgery, received isoflurane, received rocuronium muscle relaxant, and received blood product were all factors significantly associated with POCD. Having received desflurane and muscle relaxant reversal were both significantly more common among those found not to have POCD. More preoperative polypharmacy was identified in the POCD group than in the non-POCD group; however, the difference between groups did not reach statistical significance (84.3 *vs*. 71.8%, respectively; *p* = 0.080).

**Table 4:** Variables potentially associated with postoperative cognitive dysfunction (POCD) compared between the non-POCD and POCD groups.

**Variables**	**Non-POCD (*n* = 124)**	**POCD (*n* = 51)**	***p*-value**
**Preoperative data**			
Polypharmacy	89 (71.8%)	43 (84.3%)	0.080
PIMs	35 (22.7%)	14 (27.5%)	0.917
Age (years)	73.26 ± 6.46	71.10 ± 7.24	0.074
Male gender	69 (55.6%)	34 (66.7%)	0.178
ASA Status			0.061
II	34 (27.4%)	5 (9.8%)	
III	85 (68.5%)	41 (80.4%)	
IV	5 (4.0%)	5 (9.8%)	
**Comorbidity**			
CCI	5.78 ± 1.74	5.76 ± 2.04	0.954
CCI <6	61 (49.2)	26 (51.0)	
CCI ≥ 6	63 (50.8)	25 (49.0)	
Congestive heart failure	10 (8.1%)	8 (15.7%)	0.131
Ischemic heart disease/myocardial infarction	51 (41.1%)	31 (60.8%)	0.018^*^
Valvular heart disease	25 (20.2%)	17 (33.3%)	0.064
**Preoperative medication**			
Antiemetic drug	2 (1.6%)	3 (5.9%)	0.151
Antihypertensive drug	99 (79.8%)	47 (92.2%)	0.046^*^
Pre-benzodiazepine	23 (18.5%)	10 (19.6%)	0.889
Site of surgery			<0.001^*^
Non-CVT	63 (50.8%)	12 (23.5%)	
CVT	61 (49.2%)	39 (76.5%)	
**Intraoperative data**			
Choice of anesthesia			0.022^*^
GA and RA	22 (17.7%)	5 (9.8%)	
GA	86 (69.4%)	45 (88.2%)	
RA	16 (12.9%)	1 (2.0%)	
Benzodiazepine	56 (45.2%)	36 (70.6%)	0.002^*^
Inhalation use			0.442
No	23 (18.5%)	7 (13.7%)	
Yes	101 (81.5%)	44 (86.3%)	
**Inhalation type**			
Desflurane	24 (19.4%)	2 (3.9%)	0.009^*^
Isoflurane	29 (23.4%)	27 (52.9%)	<0.001^*^
**Muscle relaxant for intubation**			
Rocuronium	40 (32.3%)	27 (52.9%)	0.011^*^
Reversal agents used	41 (33.6%)	9 (17.6%)	0.035^*^
Received blood product	70 (57.4%)	41 (80.4%)	0.004^*^
**Postoperative data**			
Post-benzodiazepine	48 (39.6%)	26 (50.9%)	0.178

*Data presented as number and percentage or mean ± standard deviation*.

*A p < 0.05 indicates statistical significance*.

*POCD, postoperative cognitive dysfunction; PIMs, potentially inappropriate medications; ASA, American Society of Anesthesiologists; CVT, cardiovascular thoracic; GA, general anesthesia; RA, regional anesthesia; Pre-benzodiazepine, The patient was given benzodiazepine before the operation; Post-benzodiazepine, The patient was given benzodiazepine after the operation; CCI, charlson comorbidity index*.

* *p < 0.05*.

**Figure 3:**
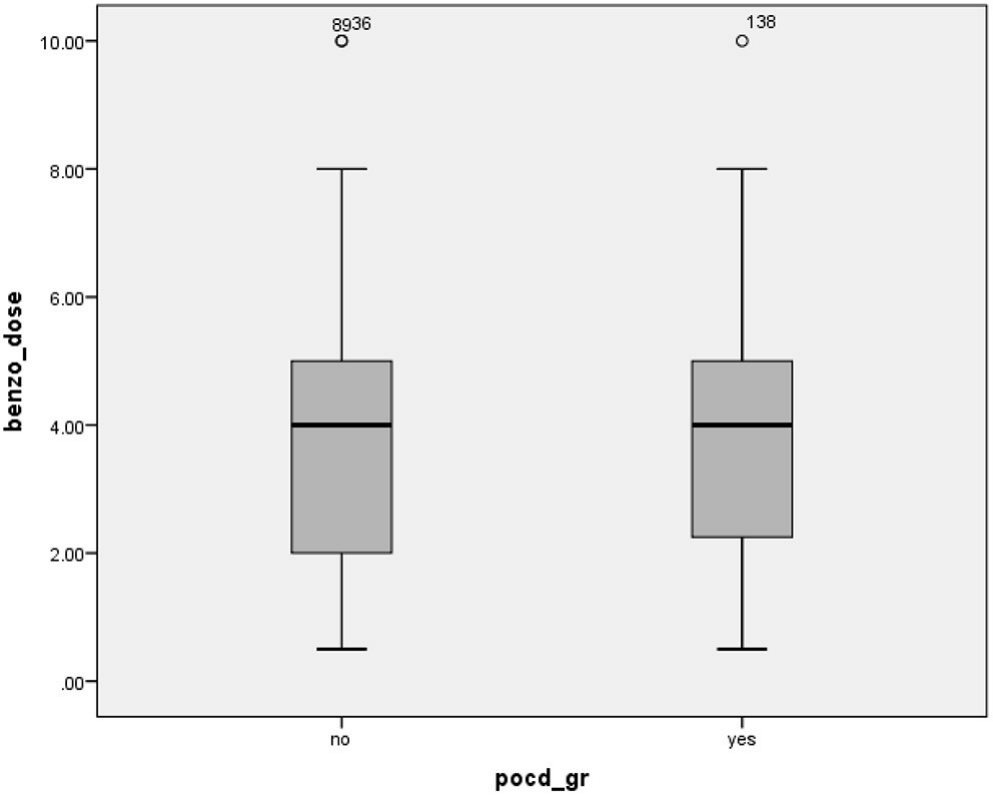
Median (IQR) dosage of intraoperative benzodiazepine compared between the non-POCD and POCD groups (*p* > 0.05). IQR, interquartile range; POCD, postoperative cognitive disorder.

Univariate analysis to identify factors that are potentially independently associated with POCD in older adult patients after major surgery is shown in Table 5. Those results show that all included variables were significantly associated with POCD, except polypharmacy and age. Univariate analysis revealed only a trend toward significant association between polypharmacy and POCD with a crude odds ratio (OR) of 2.11 (95% CI: 0.90–4.94, *p* = 0.08). The three strongest risk factors for POCD were use of an isoflurane inhaler, undergoing CVT surgery, and receiving blood product during operation with crude ORs of 3.68 (95% CI: 1.85–7.34, *p* < 0.001), 3.35 (95% CI: 1.60–7.01, *p* < 0.001), and 3.04 (95% CI: 1.39–6.63, *p* = 0.005), respectively. Interestingly, receiving desflurane was a protective factor for POCD with a crude OR of 0.17 (95% CI: 0.03–0.74, *p* = 0.019). Exploratory analysis was the carried out to identify independent predictors of POCD. Having all significant factors from univariate analysis in the multivariate model showed the effect of collinearity of the involved factors. The selection of factors to be included was based on the OR, the *p*-value, and the clinical meaningfulness of the factors according to the judgment of the authors. The final models for multivariate analysis are shown in Table 6. Since inhaler agents were found to be significantly associated with POCD in univariate analysis, the decision was made to have two models to separately explore the effect of this medication group. After adjusting for polypharmacy, perioperative benzodiazepine use remained an independent risk factor for POCD with an adjusted OR of 2.27 (95% CI: 1.10–4.68, *p* = 0.026); however, the protective effect of desflurane use was not sustained (adjusted OR [aOR]: 0.21, 95% CI: 0.47–0.95; *p* = 0.066). Model B revealed benzodiazepine and isoflurane to both be independently associated with POCD with aORs of 2.11 (95% CI: 1.00–4.43, *p* = 0.048) and 2.80 (95% CI: 1.35–5.81, *p* = 0.006), respectively. Additional analysis was performed to explore the effect of dosage of benzodiazepine between the POCD and non-POCD groups. That analysis revealed no significant difference between groups (Figure 3). Table 7 shows significantly higher benzodiazepine and isoflurane use in the polypharmacy group than in the non-polypharmacy group (both *p* < 0.05).

**Table 5:** Univariate analysis to identify factors that are potentially independently associated with postoperative cognitive dysfunction (POCD) in older adult patients after major surgery.

**Factors**	**Non-POCD (*n* = 124)**	**POCD (*n* = 51)**	**Univariate analysis**	
			**Crude OR (95% CI)**	***p***
Polypharmacy	89 (71.8%)	43 (84.3%)	2.11 (0.90–4.94)	0.084
Age	73.26 ± 6.46	71.10 ± 7.24	0.95 (0.90–1.00)	0.056
IHD/MI	51 (41.1%)	31 (60.8%)	2.21 (1.14–4.32)	0.019^*^
**Site of surgery**				
Non-CVT	63 (50.8%)	12 (23.5%)	Reference	
CVT	61 (49.2%)	39 (76.5%)	3.35 (1.60–7.01)	<0.001^*^
Benzodiazepine	56 (45.2%)	36 (70.6%)	2.91 (1.44–5.86)	0.003^*^
Desflurane	24 (19.4%)	2 (3.9%)	0.17 (0.03–0.74)	0.019^*^
Isoflurane	29 (23.4%)	27 (52.9%)	3.68 (1.85–7.34)	<0.001^*^
Rocuronium	40 (32.3%)	27 (52.9%)	2.36 (1.21–4.60)	0.011^*^
Reversal agents used	41 (33.6%)	9 (17.6%)	0.42 (0.18–0.95)	0.038^*^
Received blood product	70 (57.4%)	41 (80.4%)	3.04 (1.39–6.63)	0.005^*^

*Factors with a p < 0.10 in univariate analysis will be entered into multivariate analyses*.

*POCD, postoperative cognitive dysfunction; OR, odds ratio; CI, confidence interval; IHD/MI, ischemic heart disease/myocardial infarction; CVT, cardiovascular thoracic surgery*.

* *p < 0.05*.

**Table 6:** Multivariate analyses (models A and B) to identify independent predictors of postoperative cognitive dysfunction (POCD) in older adult patients after major surgery.

**Predictors**	**Model A (Included desflurane)**		**Model B (Included isoflurane)**	
	**Adjusted OR (95% CI)**	***p*-value**	**Adjusted OR (95% CI)**	***p*-value**
Polypharmacy	1.91 (0.92–4.62)	0.149	1.44 (0.58–3.55)	0.426
Benzodiazepine	2.27 (1.10–4.68)	0.026^*^	2.11 (1.00–4.43)	0.048^*^
Desflurane	0.21 (0.47–0.95)	0.066	–	–
Isoflurane	–	–	2.80 (1.35–5.81)	0.006^*^

*A p < 0.05 indicates statistical significance*.

*Multivariate backward stepwise logistic regression was analyzed separately for multicollinearity variables*.

*POCD, postoperative cognitive dysfunction; OR, odds ratio; CI, confidence interval*.

* *p < 0.05*.

**Table 7:** Prevalence of the independent factors that predict postoperative cognitive dysfunction (POCD) in older adult patients after major surgery compared between the non-polypharmacy and polypharmacy groups.

**Factors**	**Non-polypharmacy (*n* = 8)**	**Polypharmacy (*n* = 43)**	***p*-value**
Intraoperative benzodiazepine	3 (37.5%)	33 (76.7%)	0.039^*^
Isoflurane	1 (12.5%)	26 (60.5%)	0.019^*^

*A p < 0.05 indicates statistical significance*.

* *p < 0.05*.

## Discussion

The prevalence of polypharmacy among older adult patients who underwent major surgery in this study was very high. This study also investigated for correlation between preoperative polypharmacy and POCD, which is a clinical setting for which data remain scarce. The results of this study showed only a trend toward significant association between polypharmacy and POCD (*p* = 0.08). Interestingly, we identified other medications that are commonly used intraoperatively to be both significantly and independently associated with POCD.

Polypharmacy is very common among older adults, which is a subpopulation that frequently has more chronic comorbidities that require a larger number of drugs to manage their illnesses (25, 26). However, the prevalence of polypharmacy is under-studied and under-reported in the surgical setting. Recent studies have reported a prevalence of polypharmacy of 40–55% (9, 10, 27, 28). The negative consequences of polypharmacy in a postopearative setting have been variously reported (9, 10, 27, 28). Some studies found polypharmacy to be associated with poor postoperative outcomes, such as postoperative complications, functional decline, hospitalization, and increased mortality (10, 27); however, other studies did not report those results (9, 28). A prospective multicenter observational study (28) in older adults undergoing elective surgery found polypharmacy to be unrelated to the development of POCD. The present study found a very high 74% prevalence of polypharmacy, which is substantially higher than the rates reported in previous studies. Even though we found only a trend (*p* = 0.08) toward significant association between polypharmacy and POCD among older adult patients who underwent elective major surgery, careful monitoring of older adults with polypharmacy should still be recommended in this setting.

POCD is a postoperative phenomenon that has been increasingly studied during the last decade. Although the exact pathology of POCD is unknown, it is thought to be caused by an inflammatory process in the brain. The systemic responses induced by anesthesia and surgery may trigger neuroinflammation and subsequent POCD. Many risk factors are thought to influence POCD onset, including increasing age, poor education, a history of cerebrovascular disease with no residual impairment, the duration and type of surgery, preexisting cognitive impairment, poor functional status, multiple comorbidities and severity of illness, and postoperative respiratory complications (29, 30). In line with the finding from other studies, older adults in the polypharmacy group have a greater percentage of comorbidities. However, consider from the chalrlson's comorbiditity index between the POCD and non-POCD group, it appears that the complexity of comorbid diseases were not influence the occurence of POCD in the present study.

Our multivariate analyses showed POCD to be independently associated with intraoperative benzodiazepine and isoflurane, which are anesthetic drugs, but not with other baseline risk factors. The association between anesthetics and cognitive impairment is of both interest and concern. Many medications used during anesthesia have systemic effects, with particular effects on cognitive abilities after surgery. However, the mechanism of anesthetics in POCD remains unknown, but several mechanisms have been proposed. Factors that may contribute to POCD include anesthetic approach, monitoring modality, and intraoperative complications. These factors influence modification of the tau protein, inflammation process, calcium dysregulation, and mitochondrial dysfunction, which have all been proposed to influence postoperative cognitive impairment (13).

Benzodiazepines are categorized as delirium-inducing medications (DIMs) (31, 32). This group of drugs is contraindicated in patients at high-risk for developing delirium (33). However, the studies in the relationship between benzodiazepine and POCD are few, and the results are conflicting. Li et al. (34) conducted a prospective randomized controlled trial of 164 older adult patients who underwent hip or knee replacement under spinal-epidural anesthesia (CSE) to assess the effects of dexmedetomidine, propofol, or midazolam sedation on POCD. They found that the group given midazolam had the highest prevalence of POCD. However, another study that was conducted by Mansouri et al. (35) in 150 candidates aged over 65 years who underwent cataract surgery under general anesthesia found that patients who received midazolam had a lower incidence of POCD than those who received a placebo. A systematic review by Kok et al. (36) investigated the function of benzodiazepine as a sedative agent during ICU admission. Their results revealed benzodiazepine to be a significant risk factor for cognitive impairment during and after admission. Several pathogeneses via the GABAergic neurotransmitter system have been proposed. The independent associations identified in the present study add to the existing body of evidence regarding the short-term detrimental effect of benzodiazepine in older adult patients.

Isoflurane, sevoflurane, and desflurane are inhalation anesthetics that are essential agents for maintaining general anesthesia for longer periods. However, they can effectuate neuroinflammation, which can adversely affect the cognitive function of older adults. Nevertheless, limited studies have been conducted in human that addressed the association between specific inhalation agent and the occurrence of POCD (13). Several studies in animal models demonstrated derangement in neurotransmitters and cytokines during isoflurane administration. Acharya et al. reported that isoflurane increased blood-brain barrier (BBB) permeability by destroying brain vascular endothelial cells in aging mice, which resulted in the secretion of various cytokines and proinflammatory mediators into the brain. This combined release caused abnormal brain function, and contributed to postoperative cognitive dysfunction (37). According to the findings of Cao et al., isoflurane induces inflammatory processes by secreting a significant quantity of proinflammatory cytokines, including tumor necrosis factor (TNF), interleukin 6 (IL-6), and interleukin 1 beta (IL-1β), which resulted in the deposition of a large amount of hypoxia-inducible factor 1-alpha (HIF-1α) protein at the hippocampus. They reported these findings to be associated with cognitive impairment (38). In the laboratory, Xie et al. (39) investigated H4 human neuroglioma cells that had been exposed to isoflurane. Their results showed increased amounts of β-amyloid protein and amyloid precursor protein (APP), which are both known to be associated with Alzheimer's disease (AD). Another study in animals that was conducted by Liu et al. (40) found that isoflurane enhanced the development of Alzheimer's disease (AD) (specific to spatial memory impairment) by increasing amyloid-beta (A-beta) levels and tau phosphorylation in the hippocampus of older rats. The accumulating evidence from animal studies suggests that isoflurane exposure in older adult population may be, in part, responsible for pathogenesis of POCD observed in the present study.

Desflurane is a volatile anesthetic drug with a low blood-gas partition coefficient that provides faster recovery following general anesthesia, which is thought to protect against cognitive decline after anesthesia. The present study demonstrated a significant association effect of desflurane to reduce risk of POCD in univariate analysis; however, that significant effect was not sustained in multivariate analysis. It was reported that desflurane enhanced neurologic outcomes in patients who underwent cardiac bypass surgery, and in patients with hypoxic neuronal impairment (41). However, another study that investigated the effect of two volatile anesthetic drugs (desflurane and sevoflurane) on POCD reported no significant difference in the incidence of POCD between the two drugs (42). Given the mixed results from current and previous studies, further studies are needed to explore the effect of desflurane on POCD in older adult patients requiring anesthesia.

### Strengths and Limitations

This study has several mentionable strengths and limitations. Regarding strengths, medication reconciliation is a routine practice in our center, so our determination of the medication used could be considered reliable. Second, this study was a prospective study designed to investigate for POCD, potential confounding factors and outcome were preplanned and therefore less likely to underestimate or misclassification. Concerning limitations, the first weakness of this study is that our data were derived from a single center, the generalizability might be limited and should preferable be further explored in other setting. Second, MoCA retesting was only performed in 175 patients out of 250 subjects initially included. This might lead to bias estimation of prevalence of POCD. However, we have compared baseline characteristics of those for whom MoCA was not repeated and discovered no statistical difference with the included group. We, therefore, hypothesize that the missing data would have minimal effect on the prevalence of POCD identified in the study. However, although the sample size was sufficient to investigate the prevalence of polypharmacy, it might not be adequate for exploring the associated with POCD. Should the observed odd ratios of two from the association between polypharmacy and POCD be considered as clinically significant, it would require sample size around 400, estimated from the prevalence of polypharmacy. This study could have been underpowered to explore the association for polypharmacy and POCD. Furthermore, based on the finding of this study and a review of previous findings, it emerges that the effects of isoflurane and desflurane on POCD in humans are fascinating and could lead to changes in anesthetic practice. Further study with a larger sample size to explore the associations between those anesthetic agents, polypharmacy, and POCD is warranted.

## Conclusions

The prevalence of polypharmacy in this study was a very high 74%. There was a non-significant trend toward (*p* = 0.08) increased risk of POCD in the polypharmacy group. Intraoperative benzodiazepine and isoflurane were identified as independent predictors of POCD, whereas desflurane was found to be an independent protective factor against POCD. Since POCD can significantly adversely impact older adult patients and their families, strategies to manage these modifiable factors are needed to improve patient outcomes.

## Data Availability Statement

The raw data supporting the conclusions of this article will be made available by the authors, without undue reservation.

## Ethics Statement

The protocol for this study was approved by the Institutional Review Board of the Human Research Protection Unit, Faculty of Medicine Siriraj Hospital, Mahidol University, Bangkok, Thailand (COA No. Si 189/2019). The patients/participants provided their written informed consent to participate in this study. Written informed consent was obtained from the individual(s) for the publication of any potentially identifiable images or data included in this article.

## Author Contributions

SL, WL, NM, WW, AS, and VS contributed to the study's conception and design. SL, AS, PS, and VS were responsible for data collection and analysis. SL, WL, AS, and VS interpreted the data and prepared the manuscript. All authors read and approved the final manuscript.

## Funding

This research was funded by a grant from the Faculty of Medicine Siriraj Hospital, Mahidol University, Bangkok, Thailand (Grand Number R016231043).

## Conflict of Interest

The authors declare that the research was conducted in the absence of any commercial or financial relationships that could be construed as a potential conflict of interest.

## Publisher's Note

All claims expressed in this article are solely those of the authors and do not necessarily represent those of their affiliated organizations, or those of the publisher, the editors and the reviewers. Any product that may be evaluated in this article, or claim that may be made by its manufacturer, is not guaranteed or endorsed by the publisher.
